# Translocation of a Stray Cat Infected with Rabies from North Carolina to a Terrestrial Rabies-Free County in Ohio, 2017

**DOI:** 10.15585/mmwr.mm6742a2

**Published:** 2018-10-26

**Authors:** Amber J. Singh, Richard B. Chipman, Sietske de Fijter, Richard Gary, Marilyn G. Haskell, Jordona Kirby, Li Yu, Rene E. Condori, Lillian Orciari, Ryan Wallace

**Affiliations:** ^1^Zoonotic Disease Program, Bureau of Infectious Diseases, Ohio Department of Health; ^2^Wildlife Services, Animal and Plant Health Inspection, U.S. Department of Agriculture, Washington, DC; ^3^Epidemiology Section, Division of Public Health, North Carolina Department of Health and Human Services; ^4^Division of High-Consequence Pathogens and Pathology, National Center for Emerging Zoonotic Infectious Diseases, CDC.

On July 24, 2017, the Ohio Department of Health (ODH) was notified of a positive rabies test result from a domestic cat in Summit County, a county considered free from terrestrial rabies. Oral rabies vaccination (ORV) of raccoons, in the form of consumable bait, is conducted each year along the Ohio-Pennsylvania border to prevent the westward expansion of the raccoon rabies virus variant (RVV). In the United States, several distinct rabies virus variants exist; raccoon RVV is enzootic along the eastern parts of the United States (from Florida to Maine), including several counties in northeast Ohio (*1*). Animal rabies vaccination is protective against all rabies virus variants. The rabid cat (cat A) was located west of the ORV barrier, raising concern that it had acquired the infection from a raccoon and suggesting a possible breach in the ORV barrier (Figure 1). ODH initiated an investigation to identify persons and animals exposed to the rabid cat during its viral shedding period and collaborated with CDC to determine the likely origin of the virus (Figure 2). Public health investigators later discovered that the cat originated in North Carolina. Phylogenetic analysis confirmed that the virus was most similar to the raccoon RVV that circulates in North Carolina (Figure 3); therefore, this ORV breach was likely the result of human-mediated movement of a rabid animal rather than natural expansion of the raccoon rabies virus enzootic area. This report summarizes the investigation and highlights the importance of owner compliance regarding rabies vaccination.

**FIGURE 1:**
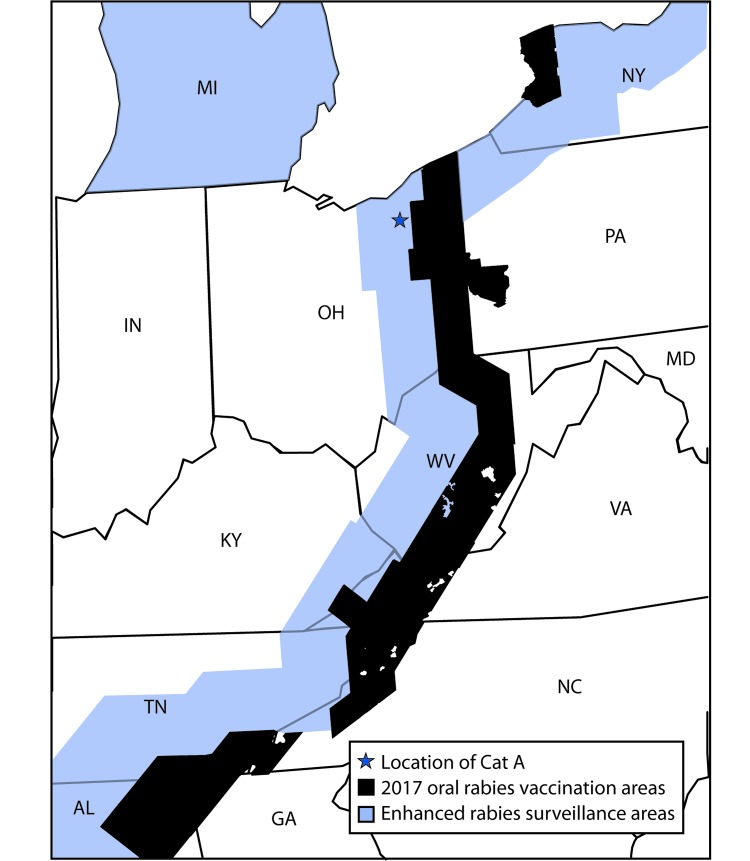
Oral rabies vaccination and enhanced rabies surveillance areas in Ohio and surrounding states, 2017 **Abbreviations:** AL = Alabama; GA = Georgia; IN = Indiana; KY = Kentucky; MD = Maryland; MI = Michigan; NC = North Carolina; NY = New York; OH = Ohio; PA = Pennsylvania; TN = Tennessee; VA = Virginia; WV = West Virginia.

**FIGURE 2:**
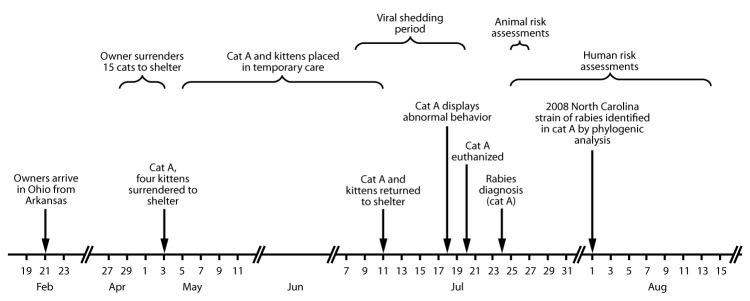
Public health investigation of a rabid cat (Cat A) translocated from North Carolina to Ohio — February–August 2017* * During human risk assessments, all potentially exposed persons were contacted by telephone. Three attempts were made by telephone and if no contact was made, a letter was sent to the residence. Three persons were lost to follow-up; 92% of the risk assessments were completed within 4 days of diagnosis of rabies in the cat. The remaining two were completed 7 days and 21 days after the diagnosis.

**FIGURE 3:**
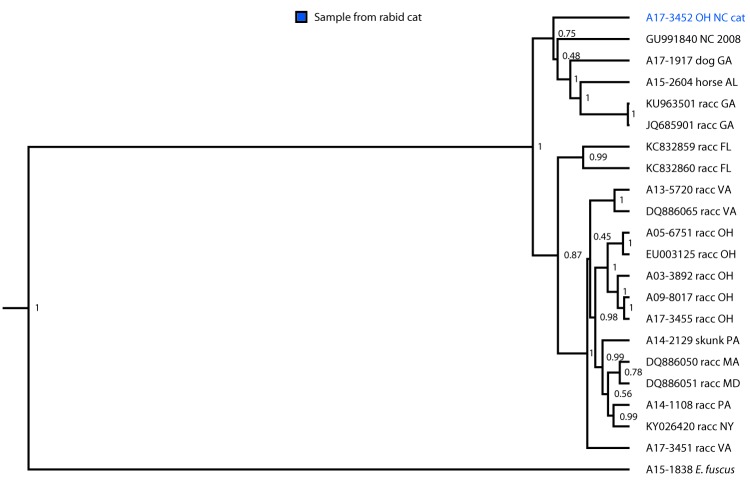
Phylogenetic analysis* of rabies virus variant from a rabid cat — Ohio, 2017 * Branch length is related to the number of nucleotide substitutions. The more substitutions, the longer the branch. More evolved variants are further from their ancestor.

## Case Report

In early February 2017, two adults traveled from North Carolina to Arkansas with two dogs and 13 cats. The stray cats (including cat A) were taken in as personal pets while the couple lived in North Carolina. They departed Arkansas on February 17, and arrived in Summit County, Ohio, for temporary residence with family members on February 21. The cats were co-housed in a garage until May 3. During that time, the cats were not permitted out of the garage, and no known exposures to other mammals occurred. While housed in the garage, cat A and another cat gave birth to an unknown number of kittens, several of which died of unknown causes.

During April 28–May 3, because of inability to care for the animals, the owner surrendered 15 cats to the Humane Society of Summit County (a local nonprofit organization), including cat A and her four kittens. Upon intake, the animals received a physical exam, routine vaccines, an antihelminthic, flea and tick control, and feline immunodeficiency virus and feline leukemia virus testing. Because the rabies vaccine is administered at time of spay/neuter at this facility, cat A did not receive a rabies vaccine. Cat A and her four kittens were placed in a temporary home during May 5–July 11 to allow the kittens to be weaned. Cat A was examined by a veterinarian on May 24, June 1, and June 9 for an upper respiratory tract infection. On July 8, cat A was reported to be “making abnormal vocalizations,” which were attributed to estrus. Upon return to the shelter on July 11, no abnormalities were noted in either cat A or the kittens. On July 18, almost 5 months after arriving in Ohio, cat A developed abnormal behavior including excessive panting, agitation, open-mouthed breathing, and hind-limb ataxia. Despite treatment, symptoms progressed rapidly and cat A was euthanized on July 20. Although no one was bitten and there was no evidence that cat A had been exposed to a rabies vector species, the cat’s head was submitted for rabies testing. The ODH laboratory reported a positive rabies result on July 24, the same day the sample was received. After consultation with CDC, a brain tissue sample was submitted for further characterization to determine the likely origin of the virus. Antigenic typing confirmed that the virus was raccoon RVV, and molecular characterization of the rabies virus N-gene determined that it was most similar to a clade found in central North Carolina, suggesting that cat A had been infected before being translocated to Ohio.

## Public Health Investigation

ODH developed a risk assessment tool to identify persons and animals potentially exposed to cat A during the viral shedding period (July 8–20), defined as 10 days before development of clinical signs (July 18) until the time of the cat’s death (July 20) (*1*). Among 29 identified persons with potential contact, 26 (90%) were located and completed the risk assessment. Postexposure prophylaxis was recommended to three humane society staff members and one adult caring for cat A in the temporary home. Conditions of exposure included known and presumed exposure to saliva such as administration of oral medications, inability to determine if a bite or scratch had occurred, and possible breach of infection control during decapitation.

The 14 remaining cats had either been adopted or placed in temporary care among 12 households. Cat A’s four kittens were presumed to be exposed because of contact with her saliva during grooming and nursing behaviors. The other 10 cats that had been co-housed in the garage with cat A were not considered exposed during the viral shedding period; however, exposure to a common source animal in North Carolina could not be ruled out, and all 10 cats were considered potentially exposed. Six of the 14 cats had not received rabies vaccine before adoption because they had been too young; owners were advised to vaccinate these animals at the appropriate age, according to veterinarian recommendations (*2*,*3*). In concordance with the 2011 Compendium of Animal Rabies Prevention and Control (*2*), all cats were placed under strict quarantine for 6 months to preclude the risk for further transmission.* Quarantine was managed either at the shelter or in their adoptive homes. As of May 7, 2018, no additional cases of rabies related to these animals were identified.

## Discussion

The United States is host to five non-bat terrestrial rabies reservoir species, each with its own genetically distinct RVV and geographic distribution. The raccoon RVV is found in 19 states in the eastern United States, but in only several counties in northeast Ohio that border Pennsylvania; Summit County is not considered enzootic for rabies RVV. The U.S. Department of Agriculture (USDA), Animal and Plant Health Inspection Service Wildlife Services commits over $20 million annually to oral rabies vaccine (ORV) programs in the eastern United States. In Ohio, the ORV zone covers several counties in the northeastern part of the state. Vaccine-laden baits are dropped in these areas to create a barrier of immunized animals that aids in reducing the number of animal rabies cases and prevents the westward spread of raccoon RVV. Enhanced rabies surveillance is conducted along the ORV zone, which includes Summit County, to ensure that ORV breaches are quickly recognized and confirmed; risk assessments are conducted to guide the necessity and scope for emergency management action. When breaches occur, emergency vaccination operations are typically conducted by USDA in collaboration with federal, state, and local partners.

Phylogenetic and epidemiologic evidence was used to rule out a breach by natural expansion; in this case, human intervention appears to have been responsible for the translocation of raccoon RVV from North Carolina (an enzootic state) into Summit County, Ohio, a county free from the viral variant. Molecular characterization of the virus variant was essential in determining the source of the virus, which guided public health intervention efforts on local, state, and federal levels. Had the evidence suggested a natural expansion event, an emergency ORV baiting operation would likely have been initiated. However, given that the translocation event was likely human-mediated, and the event was quickly contained, additional ORV baiting was deemed unnecessary.

Human-mediated translocation (movement of an animal from its home range to a new area) of rabid animals has occurred in the past. These events can have profound public health implications, as seen by the translocation of a rabid raccoon from Florida to Virginia in the 1970s and the translocation of a rabid raccoon from Vermont to Hamilton, Canada, in 2015 (*3*,*4*). Both events triggered large-scale wildlife rabies epizootics and control measures that continue to this day. CDC estimates the cost of public health expenditures on rabies disease diagnostics, prevention, and control in the United States at $245 to $510 million annually (*5*). Expansion of rabies enzootic zones, either through translocation or natural expansion, could substantially increase both the cost and the burden on public health resources.

The rabies virus isolated from this cat was determined to be associated with a viral clade found only in central North Carolina, the same location where cat A was procured as a stray. No other cats in the captured cohort were reported to have developed signs of rabies from the time the owner left North Carolina (February) up to the time of surrender in May. Therefore, cat A likely was infected with the raccoon RVV as a stray followed by a minimum 5-month incubation period, which is longer than the typical incubation period of 3 weeks to 3 months (*2*). During this time, cat A passed through eight states, three of which (Arkansas, Indiana, and Mississippi) are terrestrial-rabies–free; USDA enhanced rabies surveillance does not occur in these states. Had the cat died while passing through any of these three states, it is unlikely that, in the absence of a clear human exposure, routine rabies public health surveillance would have detected the case^†^ (*3*).

None of the 13 translocated cats had a known history of rabies vaccination. Because of the public health and agriculture risks associated with translocation of unvaccinated rabies-susceptible animals, numerous federal and state animal movement laws have been enacted. These include requirements for rabies vaccination before interstate travel and procurement of a health certificate from a veterinarian (*2*,*6*). These requirements are difficult to enforce, highlighting the importance of responsible pet ownership. It is important for stray animals to receive appropriate veterinary care, with special regard to rabies vaccination, when adopted into a home. Rabies is rare in vaccinated animals (*2*); had cat A been vaccinated upon acquisition and before interstate travel, this event could have been prevented. Phylogenetic analysis and molecular characterization of the virus variant played a major role in making an evidence-based decision that prevented a costly USDA emergency response.

**Summary**

**What is already known about this topic?**

Translocation of wildlife is responsible for spreading rabies in the United States, and has led to the introduction of new rabies variants and establishment of terrestrial rabies in areas where it was previously undetected.

**What is added by this report?**

While incubating the rabies virus, a cat potentially traveled through eight states, three of which are terrestrial rabies free. If the cat had become infectious during travel, a rabies epizootic could have occurred.

**What are the implications for public health practice?**

Local rabies vaccination laws vary greatly and animals frequently cross state lines without proper veterinary care and medical documentation. Animal rabies testing, prompt investigation, interagency collaboration, and the utilization of molecular epidemiology are important in determining proper public health interventions.

## Footnotes

* At the time of this event, the Ohio Administrative Code followed the 2011 Compendium of Animal Rabies Prevention and Control for quarantine recommendations.

† Ohio Administrative Code (OAC) 901:1–17–01: Importation and Health of Animals, General requirements; OAC 901:1–17–05: Dogs and Cats (http://codes.ohio.gov/oac/901%3A1-17); OAC 3701–3-29: Biting animal to be confined (http://codes.ohio.gov/oac/3701-3).
