# *Erratum:* Vol. 67, No. 42

**DOI:** 10.15585/mmwr.mm6744a8

**Published:** 2018-11-09

In the report “Translocation of a Stray Cat Infected with Rabies from North Carolina to a Terrestrial Rabies-Free County in Ohio, 2017,” on page 1176, the first complete sentence on that page (second sentence of the discussion) should have read “The raccoon RVV is found in 19 states in the eastern United States, but in only several counties in northeast Ohio that border Pennsylvania; Summit County is not considered enzootic for **raccoon** RVV.”

